# Metagenomics - a guide from sampling to data analysis

**DOI:** 10.1186/2042-5783-2-3

**Published:** 2012-02-09

**Authors:** Torsten Thomas, Jack Gilbert, Folker Meyer

**Affiliations:** 1 School of Biotechnology and Biomolecular Sciences & Centre for Marine Bio-Innovation, The University of New South Wales, Sydney, NSW 2052, Australia; 2 Argonne National Laboratory, 9700 South Cass Avenue, Argonne, IL 60439, USA; 3 Department of Ecology and Evolution, University of Chicago, 5640 South Ellis Avenue, Chicago, IL 60637, USA; 4 Computation Institute, University of Chicago, 5640 South Ellis Avenue, Chicago, IL 60637, USA

**Keywords:** sampling, sequencing, assembly, binning, annotation, data storage, data sharing, DNA extraction, microbial ecology, microbial diversity

## Abstract

Metagenomics applies a suite of genomic technologies and bioinformatics tools to directly access the genetic content of entire communities of organisms. The field of metagenomics has been responsible for substantial advances in microbial ecology, evolution, and diversity over the past 5 to 10 years, and many research laboratories are actively engaged in it now. With the growing numbers of activities also comes a plethora of methodological knowledge and expertise that should guide future developments in the field. This review summarizes the current opinions in metagenomics, and provides practical guidance and advice on sample processing, sequencing technology, assembly, binning, annotation, experimental design, statistical analysis, data storage, and data sharing. As more metagenomic datasets are generated, the availability of standardized procedures and shared data storage and analysis becomes increasingly important to ensure that output of individual projects can be assessed and compared.

## Introduction

Arguably, one of the most remarkable events in the field of microbial ecology in the past decade has been the advent and development of metagenomics. Metagenomics is defined as the direct genetic analysis of genomes contained with an environmental sample. The field initially started with the cloning of environmental DNA, followed by functional expression screening [1], and was then quickly complemented by direct random shotgun sequencing of environmental DNA [2,3]. These initial projects not only showed proof of principle of the metagenomic approach, but also uncovered an enormous functional gene diversity in the microbial world around us [4].

Metagenomics provides access to the functional gene composition of microbial communities and thus gives a much broader description than phylogenetic surveys, which are often based only on the diversity of one gene, for instance the 16S rRNA gene. On its own, metagenomics gives genetic information on potentially novel biocatalysts or enzymes, genomic linkages between function and phylogeny for uncultured organisms, and evolutionary profiles of community function and structure. It can also be complemented with metatranscriptomic or metaproteomic approaches to describe expressed activities [5,6]. Metagenomics is also a powerful tool for generating novel hypotheses of microbial function; the remarkable discoveries of proteorhodopsin-based photoheterotrophy or ammonia-oxidizing Archaea attest to this fact [7,8].

The rapid and substantial cost reduction in next-generation sequencing has dramatically accelerated the development of sequence-based metagenomics. In fact, the number of metagenome shotgun sequence datasets has exploded in the past few years. In the future, metagenomics will be used in the same manner as 16S rRNA gene fingerprinting methods to describe microbial community profiles. It will therefore become a standard tool for many laboratories and scientists working in the field of microbial ecology.

**Figure 1:**
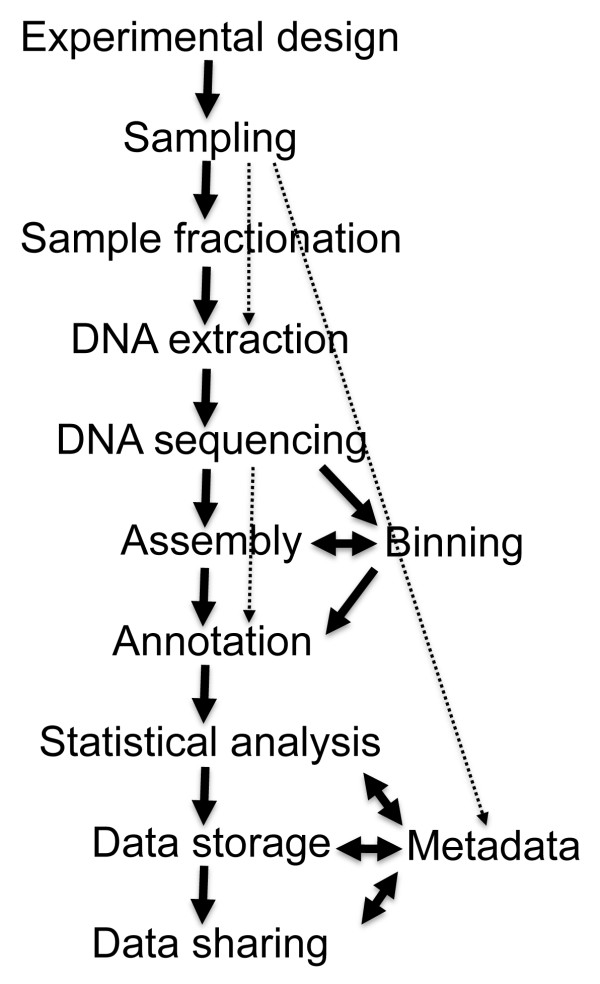
**Flow diagram of a typical metagenome projects**. Dashed arrows indicate steps that can be omitted.

This review gives an overview of the field of metagenomics, with particular emphasis on the steps involved in a typical sequence-based metagenome project (Figure 1). We describe and discuss sample processing, sequencing technology, assembly, binning, annotation, experimental design, statistical analysis, and data storage and sharing. Clearly, any kind of metagenomic dataset will benefit from the rich information available from other metagenome projects, and it is hoped that common, yet flexible, standards and interactions among scientists in the field will facilitate this sharing of information. This review article summarizes the current thinking in the field and introduces current practices and key issues that those scientists new to the field need to consider for a successful metagenome project.

## Sampling and processing

Sample processing is the first and most crucial step in any metagenomics project. The DNA extracted should be representative of all cells present in the sample and sufficient amounts of high-quality nucleic acids must be obtained for subsequent library production and sequencing. Processing requires specific protocols for each sample type, and various robust methods for DNA extraction are available (e.g. [3,9,10]). Initiatives are also under way to explore the microbial biodiversity from tens of thousands of ecosystems using a single DNA extraction technology to ensure comparability [11].

If the target community is associated with a host (e.g. an invertebrate or plant), then either fractionation or selective lysis might be suitable to ensure that minimal host DNA is obtained (e.g. [9,12]). This is particularly important when the host genome is large and hence might "overwhelm" the sequences of the microbial community in the subsequent sequencing effort. Physical fractionation is also applicable when only a certain part of the community is the target of analysis, for example, in viruses seawater samples. Here a range of selective filtration or centrifugation steps, or even flow cytometry, can be used to enrich the target fraction [3,13,14]. Fractionation steps should be checked to ensure that sufficient enrichment of the target is achieved and that minimal contamination of non-target material occurs.

Physical separation and isolation of cells from the samples might also be important to maximize DNA yield or avoid coextraction of enzymatic inhibitors (such as humic acids) that might interfere with subsequent processing. This situation is particularly relevant for soil metagenome projects, and substantial work has been done in this field to address the issue ([10] and references therein). Direct lysis of cells in the soil matrix versus indirect lysis (i.e. after separation of cells from the soil) has a quantifiable bias in terms of microbial diversity, DNA yield, and resulting sequence fragment length [10]. The extensive work on soil highlights the need to ensure that extraction procedures are well benchmarked and that multiple methods are compared to ensure representative extraction of DNA.

Certain types of samples (such as biopsies or ground-water) often yield only very small amounts of DNA [15]. Library production for most sequencing technologies require high nanograms or micrograms amounts of DNA (see below), and hence amplification of starting material might be required. Multiple displacement amplification (MDA) using random hexamers and phage phi29 polymerase is one option employed to increase DNA yields. This method can amplify femtograms of DNA to produce micrograms of product and thus has been widely used in single-cell genomics and to a certain extent in metagenomics [16,17]. As with any amplification method, there are potential problems associated with reagent contaminations, chimera formation and sequence bias in the amplification, and their impact will depend on the amount and type of starting material and the required number of amplification rounds to produce sufficient amounts of nucleic acids. These issues can have significant impact on subsequent metagenomic community analysis [15], and so it will be necessary to consider whether amplification is permissible.

## Sequencing technology

Over the past 10 years metagenomic shotgun sequencing has gradually shifted from classical Sanger sequencing technology to next-generation sequencing (NGS). Sanger sequencing, however, is still considered the gold standard for sequencing, because of its low error rate, long read length (> 700 bp) and large insert sizes (e.g. > 30 Kb for fosmids or bacterial artificial chromosomes (BACs)). All of these aspects will improve assembly outcomes for shotgun data, and hence Sanger sequencing might still be applicable if generating close-to-complete genomes in low-diversity environments is the objective [18]. A drawback of Sanger sequencing is the labor-intensive cloning process in its associated bias against genes toxic for the cloning host [19] and the overall cost per gigabase (appr. USD 400,000).

Of the NGS technologies, both the 454/Roche and the Illumina/Solexa systems have now been extensively applied to metagenomic samples. Excellent reviews of these technologies are available [20,21], but a brief summary is given here with particular attention to metagenomic applications.

The 454/Roche system applies emulsion polymerase chain reaction (ePCR) to clonally amplify random DNA fragments, which are attached to microscopic beads. Beads are deposited into the wells of a picotitre plate and then individually and in parallel pyrosequenced. The pyrosequencing process involves the sequential addition of all four deoxynucleoside triphosphates, which, if complementary to the template strand, are incorporated by a DNA polymerase. This polymerization reaction releases pyrophosphate, which is converted via two enzymatic reactions to produce light. Light production of ~ 1.2 million reactions is detected in parallel via a charge-coupled device (CCD) camera and converted to the actual sequence of the template. Two aspects are important in this process with respect to metagenomic applications. First, the ePCR has been shown to produce artificial replicate sequences, which will impact any estimates of gene abundance. Understanding the amount of replicate sequences is crucial for the data quality of sequencing runs, and replicates can be identified and filtered out with bioinformatics tools [22,23]. Second, the intensity of light produced when the polymerase runs through a homopolymer is often difficult to correlate to the actual number of nucleotide positions. Typically, this results in insertion or deletion errors in homopolymers and can hence cause reading frameshifts, if protein coding sequences (CDSs) are called on a single read. This type of error can however be incorporated into models of CDS prediction thus resulting in high, albeit not perfect, accuracy [24]. Despite these disadvantages, the much cheaper cost of ~ USD 20,000 per gigabase pair has made 454/Roche pyrosequencing a popular choice for shotgun-sequencing metagenomics. In addition, the 454/Roche technology produces an average read length between 600-800 bp, which is long enough to cause only minor loss in the number of reads that can be annotated [25]. Sample preparation has also been optimized so that tens of nanograms of DNA are sufficient for sequencing single-end libraries [26,27], although pair-end sequencing might still require micrograms quantities. Moreover, the 454/Roche sequencing platform offers multiplexing allowing for up to 12 samples to be analyzed in a single run of ~500 Mbp.

The Illumina/Solexa technology immobilizes random DNA fragments on a surface and then performs solid-surface PCR amplification, resulting in clusters of identical DNA fragments. These are then sequenced with reversible terminators in a sequencing-by-synthesis process [28]. The cluster density is enormous, with hundreds of millions of reads per surface channel and 16 channels per run on the HiSeq2000 instrument. Read length is now approaching 150 bp, and clustered fragments can be sequenced from both ends. Continuous sequence information of nearly 300 bp can be obtained from two overlapping 150 bp paired-reads from a single insert. Yields of ~60 Gbp can therefore be typically expected in a single channel. While Illumina/Solexa has limited systematic errors, some datasets have shown high error rates at the tail ends of reads [29]. In general, clipping reads has proven to be a good strategy for eliminating the error in "bad" datasets, however, sequence quality values should also be used to detect "bad" sequences. The lower costs of this technology (~ USD 50 per Gbp) and recent success in its application to metagenomics, and even the generation of draft genomes from complex dataset [30,31], are currently making the Illumina technology an increasingly popular choice. As with 454/Roche sequencing, starting material can be as low as a 20 nanograms, but larger amounts (500-1000 ng) are required when matepair-libraries for longer insert libraries are made. The limited read length of the Illumina/Solexa technology means that a greater proportion of unassembled reads might be too short for functional annotation than are with 454/Roche technology [25]. While assembly might be advisable in such a case, potential bias, such as the suppression of low-abundance species (which can not be assembled) should be considered, as should the fact that some current software packages (e.g. MG-RAST) are capable of analyzing unassembled Illumina reads of 75 bp and longer. Multiplexing of samples is also available for individual sequencing channels, with more than 500 samples multiplexed per lane. Another important factor to consider is run time, with a 2 × 100 bp paired-end sequencing analysis taking approx. 10 days HiSeq2000 instrument time, in contrast to 1 day for the 454/ Roche technology. However, faster runtime (albeit at higher cost per Gbp of approx. USD 600) can be achieved with the new Illumina MiSeq instrument. This smaller version of Illumina/Solexa technology can also be used to test-run sequencing libraries, before analysis on HiSeq instrument for deeper sequencing.

A few additional sequencing technologies are available that might prove useful for metagenomic applications, now or in the near future. The Applied Biosystems SOLiD sequencer has been extensively used, for example, in genome resequencing [32]. SOLiD arguably provides the lowest error rate of any current NGS sequencing technology, however it does not achieve reliable read length beyond 50 nucleotides. This will limit its applicability for direct gene annotation of unassembled reads or for assembly of large contigs. Nevertheless, for assembly or mapping of metagenomic data against a reference genome, recent work showed encouraging outcomes [33]. Roche is also marketing a smaller-scale sequencer based on pyrosequencing with about 100 Mbp output and low per run costs. This system might be useful, because relatively low coverage of metagenomes can establish meaningful gene profiles [34]. Ion Torrent (and more recently Ion Proton) is another emerging technology and is based on the principle that protons released during DNA polymerization can detect nucleotide incorporation. This system promises read lengths of > 100 bp and throughput on the order of magnitude of the 454/Roche sequencing systems. Pacific Biosciences (PacBio) has released a sequencing technology based on single-molecule, real-time detection in zero-mode waveguide wells. Theoretically, this technology on its RS1 platform should provide much greater read lengths than the other technologies mentioned, which would facilitate annotation and assembly. In addition, a process called strobing will mimic pair-end reads. However, accuracy of single reads with PacBio is currently only at 85%, and random reads are "dropped," making the instrument unusable in its current form for metagenomic sequencing [35]. Complete Genomics is offering a technology based on sequencing DNA nanoballs with combinatorial probe-anchor ligation [36]. Its read length of 35 nucleotides is rather limited and so might be its utility for *de novo *assemblies. While none of the emerging sequencing technologies have been thoroughly applied and tested with metagenomics samples, they offer promising alternatives and even further cost reduction.

## Assembly

If the research aims at recovering the genome of uncultured organisms or obtain full-length CDS for subsequent characterization rather than a functional description of the community, then assembly of short read fragments will be performed to obtain longer genomic contigs. The majority of current assembly programs were designed to assemble single, clonal genomes and their utility for complex pan-genomic mixtures should be approached with caution and critical evaluation.

Two strategies can be employed for metagenomics samples: reference-based assembly (co-assembly) and *de novo *assembly.

Reference-based assembly can be done with software packages such as Newbler (Roche), AMOS http://sourceforge.net/projects/amos/, or MIRA [37]. These software packages include algorithms that are fast and memory-efficient and hence can often be performed on laptop-sized machines in a couple of hours. Reference-based assembly works well, if the metagenomic dataset contains sequences where closely related reference genomes are available. However, differences in the true genome of the sample to the reference, such as a large insertion, deletion, or polymorphisms, can mean that the assembly is fragmented or that divergent regions are not covered.

*De novo *assembly typically requires larger computational resources. Thus, a whole class of assembly tools based on the de Bruijn graphs was specifically created to handle very large amounts of data [38,39]. Machine requirements for the de Bruijn assemblers Velvet [40] or SOAP [41] are still significantly higher than for reference-based assembly (co-assembly), often requiring hundreds of gigabytes of memory in a single machine and run times frequently being days.

The fact that most (if not all) microbial communities include significant variation on a strain and species level makes the use of assembly algorithms that assume clonal genomes less suitable for metagenomics. The "clonal" assumptions built into many assemblers might lead to suppression of contig formation for certain heterogeneous taxa at specific parameter settings. Recently, two de Bruijn-type assemblers, MetaVelvet and Meta-IDBA [42] have been released that deal explicitly with the non-clonality of natural populations. Both assemblers aim to identify within the entire de Bruijn graph a sub-graph that represents related genomes. Alternatively, the metagenomic sequence mix can be partition into "species bins" via k-mer binning (Titus Brown, personal communications). Those subgraphs or subsets are then resolved to build a consensus sequence of the genomes. For Meta-IDBA a improvement in terms of N50 and maximum contig length has been observed when compared to "classical" de Bruijn assembler (e.g. Velvet or SOAP; results from the personal experience of the authors; data not shown here). The development of "metagenomic assemblers" is however still at an early stage, and it is difficult to access their accuracy for real metagenomic data as typically no references exist to compare the results to. A true gold standard (i.e. a real dataset for a diverse microbial community with known reference sequences) that assemblers can be evaluated against is thus urgently required.

Several factors need to be considered when exploring the reasons for assembling metagenomic data; these can be condensed to two important questions. First, what is the length of the sequencing reads used to generate the metagenomic dataset, and are longer sequences required for annotation? Some approaches, e.g. IMG/M, prefer assembled contigs, other pipelines such as MG-RAST [43] require only 75 bp or longer for gene prediction or similarity analysis that provides taxonomic binning and functional classification. On the whole, however, the longer the sequence information, the better is the ability to obtain accurate information. One obvious impact is on annotation: the longer the sequence, the more information provided, making it easier to compare with known genetic data (e.g. via homology searches [25]). Annotation issues will be discussed in the next section. Binning and classification of DNA fragments for phylogenetic or taxonomic assignment also benefits from long, contiguous sequences and certain tools (e.g. Phylopythia) work reliably only over a specific cut-off point (e.g. 1 Kb) [44]. Second, is the dataset assembled to reduce data-processing requirements? Here, as an alternative to assembling reads into contigs, clustering near-identical reads with cd-hit [45] or uclust [46] will provide clear benefits in data reduction. The MG-RAST pipeline also uses clustering as a data reduction strategy.

Fundamentally, assembly is also driven by the specific problem that single reads have generally lower quality and hence lower confidence in accuracy than do multiple reads that cover the same segment of genetic information. Therefore, merging reads increases the quality of information. Obviously in a complex community with low sequencing depth or coverage, it is unlikely to actually get many reads that cover the same fragment of DNA. Hence assembly may be of limited value for metagenomics.

Unfortunately, without assembly, longer and more complex genetic elements (e.g., CRISPRS) cannot be analyzed. Hence there is a need for metagenomic assembly to obtain high-confidence contigs that enable the study of, for example, major repeat classes. However, none of the current assembly tools is bias-free. Several strategies have been proposed to increase assembly accuracy [38], but strategies such as removal of rare k-mers are no longer considered adequate, since rare k-mers do not represent sequence errors (as initially assumed), but instead represent reads from less abundant pan-genomes in the metagenomic mix.

## Binning

Binning refers to the process of sorting DNA sequences into groups that might represent an individual genome or genomes from closely related organisms. Several algorithms have been developed, which employ two types of information contained within a given DNA sequence. Firstly, compositional binning makes use of the fact that genomes have conserved nucleotide composition (e.g. a certain GC or the particular abundance distribution of k-mers) and this will be also reflected in sequence fragments of the genomes. Secondly, the unknown DNA fragment might encode for a gene and the similarity of this gene with known genes in a reference database can be used to classify and hence bin the sequence.

Compositional-based binning algorithms include Phylopythia [44], S-GSOM [47], PCAHIER [48,49] and TACAO [49], while examples of purely similarity-based binning software include IMG/M [50], MG-RAST [43], MEGAN [51], CARMA [52], SOrt-ITEMS [53] and MetaPhyler [54]. There is also number of binning algorithms that consider both composition and similarity, including the programs PhymmBL [55] and MetaCluster [56]. All these tools employ different methods of grouping sequences, including self-organising maps (SOMs) or hierarchical clustering, and are operated in either an unsupervised manner or with input from the user (supervised) to define bins.

Important considerations for using any binning algorithm are the type of input data available and the existence of a suitable training datasets or reference genomes. In general, composition-based binning is not reliable for short reads, as they do not contain enough information. For example, a 100 bp read can at best possess only less than half of all 256 possible 4-mers and this is not sufficient to determine a 4-mer distribution that will reliably relate this read to any other read. Compositional assignment can however be improved, if training datasets (e.g. a long DNA fragment of known origin) exist that can be used to define a compositional classifier [44]. These "training" fragments can either be derived from assembled data or from sequenced fosmids and should ideally contain a phylogenetic marker (such as a rRNA gene) that can be used for high-resolution, taxonomic assignment of the binned fragments [57].

Short reads may contain similarity to a known gene and this information can be used to putatively assign the read to a specific taxon. This taxonomic assignment obviously requires the availability of reference data. If the query sequence is only distantly related to known reference genomes, only a taxonomic assignment at a very high level (e.g. phylum) is possible. If the metagenomic dataset, however, contains two or more genomes that would fall into this high taxon assignment, then "chimeric" bins might be produced. In this case, the two genomes might be separated by additional binning based on compositional features. In general, however this might again require that the unknown fragments have a certain length.

Binning algorithm will obviously in the future benefit from the availability of a greater number and phylogenetic breadth of reference genomes, in particular for similarity-based assignment to low taxonomic levels. Post-assembly the binning of contigs can lead to the generation of partial genomes of yet-uncultured or unknown organisms, which in turn can be used to perform similarity-based binning of other metagenomic datasets. Caution should however been taken to ensure the validity of any newly created genome bin, as "contaminating" fragments can rapidly propagate into false assignments in subsequent binning efforts. Prior to assembly with clonal assemblers binning can be used to reduce the complexity of an assembly effort and might reduce computational requirement.

As major annotation pipelines like IMG/M or MG-RAST also perform taxonomic assignments of reads, one needs to carefully weigh the additional computational demands of the particular binning algorithm chosen against the added value they provide.

## Annotation

For the annotation of metagenomes two different initial pathways can be taken. First, if reconstructed genomes are the objective of the study and assembly has produced large contigs, it is preferable to use existing pipelines for genome annotation, such as RAST [58] or IMG [59]. For this approach to be successful, minimal contigs length of 30,000 bp or longer are required. Second, annotation can be performed on the entire community and relies on unassembled reads or short contigs. Here the tools for genome annotation are significantly less useful than those specifically developed for metagenomic analyses. Annotation of metagenomic sequence data has in general two steps. First, features of interest (genes) are identified (feature prediction) and, second, putative gene functions and taxonomic neighbors are assigned (functional annotation).

Feature prediction is the process of labeling sequences as genes or genomic elements. For completed genome sequences a number of algorithms have been developed [60,61] that identify CDS with more than 95% accuracy and a low false negative ratio. A number of tools were specifically designed to handle metagenomic prediction of CDS, including FragGeneScan [24], MetaGeneMark [62], MetaGeneAnnotator (MGA)/ Metagene [63] and Orphelia [64,65]. All of these tools use internal information (e.g. codon usage) to classify sequence stretches as either coding or non-coding, however they distinguish themselves from each other by the quality of the training sets used and their usefulness for short or error-prone sequences. FragGeneScan is currently the only algorithm known to the authors that explicitly models sequencing errors and thus results in gene prediction errors of only 1-2%. True positive rates of FragGeneScan are around 70% (better than most other methods), which means that even this tool still misses a significant subset of genes. These missing genes can potentially be identified by BLAST-based searches, however the size of current metagenomic datasets makes this computational expensive step often prohibitive.

There exists also a number of tools for the prediction of non-protein coding genes such as tRNAs [66,67], signal peptides [68] or CRISPRs [69,70], however they might require significant computational resources or long contiguous sequences. Clearly subsequent analysis depends on the initial identification of features and users of annotation pipelines need to be aware of the specific prediction approaches used. MG-RAST uses a two-step approach for feature identification, FGS and a similarity search for ribosomal RNAs against a non-redundant integration of the SILVA [71], Greengenes [72] and RDP [73] databases. CAMERA's RAMCAPP pipeline [74] uses FGA and MGA, while IMG/M employs a combination of tools, including FGS and MGA [58,59].

Functional annotation represents a major computational challenge for most metagenomic projects and therefore deserves much attention now and over the next years. Current estimates are that only 20 to 50% of a metagenomic sequences can be annotated [75], leaving the immediate question of importance and function of the remaining genes. We note that annotation is not done *de novo*, but via mapping to gene or protein libraries with existing knowledge (i.e., a non-redundant database). Any sequences that cannot be mapped to the known sequence space are referred to as ORFans. These ORFans are responsible for the seemingly never-ending genetic novelty in microbial metagenomics (e.g. [76]. Three hypotheses exist for existence of this unknown fraction. First, ORFans might simply reflect erroneous CDS calls caused by imperfect detection algorithms. Secondly, these ORFans are real genes, but encode for unknown biochemical functions. Third, ORFan genes have no sequence homology with known genes, but might have structural homology with known proteins, thus representing known protein families or folds. Future work will likely reveal that the truth lies somewhere between these hypotheses [77]. For improving the annotation of ORFan genes, we will rely on the challenging and labor-intensive task of protein structure analysis (e.g. via NMR and x-ray crystallography) and on biochemical characterization.

Currently, metagenomic annotation relies on classifying sequences to known functions or taxonomic units based on homology searches against available "annotated" data. Conceptually, the annotation is relatively simple and for small datasets (< 10,000 sequences) manual curation can be used increase the accuracy of any automated annotation. Metagenomic datasets are typically very large, so manual annotation is not possible. Automated annotation therefore has to become more accurate and computationally inexpensive. Currently, running a BLASTX similarity search is computationally expensive; as much as ten times the cost of sequencing [78]. Unfortunately, computationally less demanding methods involving detecting feature composition in genes [44] have limited success for short reads. With growing dataset sizes, faster algorithms are urgently needed, and several programs for similarity searches have been developed to resolve this issue [46,79-81].

Many reference databases are available to give functional context to metagenomic datasets, such as KEGG [82], eggNOG [83], COG/KOG [84], PFAM [85], and TIGRFAM [86]. However, since no reference database covers all biological functions, the ability to visualize and merge the interpretations of all database searches within a single framework is important, as implemented in the most recent versions of MG-RAST and IMG/M. It is essential that metagenome analysis platforms be able to share data in ways that map and visualize data in the framework of other platforms. These metagenomic exchange languages should also reduce the burden associated with re-processing large datasets, minimizing, the redundancy of searching and enabling the sharing of annotations that can be mapped to different ontologies and nomenclatures, thereby allowing multifaceted interpretations. The Genomic Standards Consortium (GSC) with the M5 project is providing a prototypical standard for exchange of computed metagenome analysis results, one cornerstone of these exchange languages.

Several large-scale databases are available that process and deposit metagenomic datasets. MG-RAST, IMG/M, and CAMERA are three prominent systems [43,50,74]. MG-RAST is a data repository, an analysis pipeline and a comparative genomics environment. Its fully automated pipeline provides quality control, feature prediction and functional annotation and has been optimized for achieving a trade-off between accuracy and computational efficiency for short reads using BLAT {Kent, 2002 #64}. Results are expressed in the form of abundance profiles for specific taxa or functional annotations. Supported are the comparison of NCBI taxonomies derived from 16S rRNA gene or whole genome shotgun data and the comparison of relative abundance for KEGG, eggNOG, COG and SEED subsystems on multiple levels of resolution. Users can also download all data products generated by MG-RAST, share them and publish within the portal. The MG-RAST web interface allows comparison using a number of statistical techniques and allows for the incorporation of metadata into the statistics. MG-RAST has more than 7000 users, > 38,000 uploaded and analyzed metagenomes (of which 7000 are publicly accessible) and 9 Terabases analyzed as of December 2011. These statistics demonstrate a move by the scientific community to centralize resources and standardize annotation.

IMG/M also provides a standardized pipeline, but with "higher" sensitivity as it performs, for example, hidden Markov model (HMM) and BLASTX searches at substantial computational cost. In contrast to MG-RAST, comparisons in IMG/M are not performed on an abundance table level, but are based on an all vs. all genes comparison. Therefore IMG/M is the only system that integrates all datasets into a single protein level abstraction. Both IMG/M and MG-RAST provide the ability to use stored computational results for comparison, enabling comparison of novel metagenomes with a rich body of other datasets without requiring the end-user to provide the computational means for reanalysis of all datasets involved in their study. Other systems, such as CAMERA [74], offer more flexible annotation schema but require that individual researchers understand the annotation of data and analytical pipelines well enough to be confident in their interpretation. Also for comparison, all datasets need to be analyzed using the same workflow, thus adding additional computational requirements. CAMERA allows the publication of datasets and was the first to support the Genomic Standards Consortium's Minimal Information checklists for metadata in their web interface [87].

MEGAN is another tool used for visualizing annotation results derived from BLAST searches in a functional or taxonomic dendrogram [51]. The use of dendrograms to display metagenomic data provides a collapsible network of interpretation, which makes analysis of particular functional or taxonomic groups visually easy.

## Experimental Design and Statistical Analysis

Owing to the high costs, many of the early metagenomic shotgun-sequencing projects were not replicated or were focused on targeted exploration of specific organisms (e.g. uncultured organisms in low-diversity acid mine drainage [2]). Reduction of sequencing cost (see above) and a much wider appreciation of the utility of metagenomics to address fundamental questions in microbial ecology now require proper experimental designs with appropriate replication and statistical analysis. These design and statistical aspects, while obvious, are often not properly implemented in the field of microbial ecology [88]. However, many suitable approaches and strategies are readily available from the decades of research in quantitative ecology of higher organisms (e.g. animals, plants). In a simplistic way, the data from multiple metagenomic shotgun-sequencing projects can be reduced to tables, where the columns represent samples and the rows indicate either a taxonomic group or a gene function (or groups thereof) and the fields containing abundance or presence/absence data. This is analogous to species-sample matrices in ecology of higher organisms, and hence many for the statistical tools available to identify correlations and statistically significant patterns are transferable. As metagenomic data however often contain many more species or gene functions then the number of samples taken, appropriate corrections for multiple hypothesis testing have to be implemented (e.g. Bonferroni correction for t-test based analyses).

The Primer-E package [89] is a well-established tool, allowing for a range of multivariate statistical analyses, including the generation of multidimensional scaling (MDS) plots, analysis of similarities (ANOSIM), and identification of the species or functions that contribute to the difference between two samples (SIMPER). Recently, multivariate statistics was also incorporated in a web-based tools called Metastats [90], which revealed with high confidence discriminatory functions between the replicated metagenome dataset of the gut microbiota of lean and obese mice [91]. In addition, the ShotgunFunctionalizeR package provides several statistical procedures for assessing functional differences between samples, both for individual genes and for entire pathways using the popular R statistical package [92].

Ideally, and in general, experimental design should be driven by the question asked (rather than technical or operational restriction). For example, if a project aims to identify unique taxa or functions in a particular habitat, then suitable reference samples for comparison should be taken and processed in consistent manner. In addition, variation between sample types can be due to true biological variation, (something biologist would be most interested in) and technical variation and this should be carefully considered when planning the experiment. One should also be aware that many microbial systems are highly dynamic, so temporal aspects of sampling can have a substantial impact on data analysis and interpretation. While the question of the number of replicates is often difficult to predict prior to the final statistical analysis, small-scale experiments are often useful to understand the magnitude of variation inherent in a system. For example, a small number of samples could be selected and sequenced to shallower depth, then analyzed to determine if a larger sampling size or greater sequencing effort are required to obtain statistically meaningful results [88]. Also, the level at which replication takes place is something that should not lead to false interpretation of the data. For example, if one is interested in the level of functional variation of the microbial community in habitat A, then multiple samples from this habitat should be taken and processed completely separately, but in the same manner. Taking just one sample and splitting it up prior to processing will provide information only about technical, but not biological, variation in habitat A. Taking multiple samples and then pooling them will lose all information on variability and hence will be of little use for statistical purposes. Ultimately, good experimental design of metagenomic projects will facilitate integration of datasets into new or existing ecological theories [93].

As metagenomics gradually moves through a range of explorative biodiversity surveys, it will also prove itself extremely valuable for manipulative experiments. These will allow for observation of treatment impact on the functional and phylogenetic composition of microbial communities. Initial experiments already showed promising results [94]. However, careful experimental planning and interpretations should be paramount in this field.

One of the ultimate aims of metagenomics is to link functional and phylogenetic information to the chemical, physical, and other biological parameters that characterize an environment. While measuring all these parameters can be time-consuming and cost-intensive, it allows retrospective correlation analysis of metagenomic data that was perhaps not part of the initial aim of the project or might be of interest for other research questions. The value of such metadata cannot be overstated and, in fact, has become mandatory or optional for deposition of metagenomic data into some databases [50,74].

## Sharing and Storage of Data

Data sharing has a long tradition in the field of genome research, but for metagenomic data this will require a whole new level of organization and collaboration to provide metadata and centralized services (e.g., IMG/M, CAMERA and MG-RAST) as well as sharing of both data and computational results. In order to enable sharing of computed results, some aspects of the various analytical pipelines mentioned above will need to be coordinated - a process currently under way under the auspices of the GSC. Once this has been achieved, researchers will be able to download intermediate and processed results from any one of the major repositories for local analysis or comparison.

A suite of standard languages for metadata is currently provided by the **M**inimum **I**nformation about any (**x**) **S**equence checklists (MIxS) [95]. MIxS is an umbrella term to describe MIGS (the Minimum Information about a Genome Sequence), MIMS (the Minimum Information about a Metagenome Sequence) and MIMARKS (Minimum Information about a MARKer Sequence)[87] and contains standard formats for recording environmental and experimental data. The latest of these checklists, MIMARKS builds on the foundation of the MIGS and MIMS checklists, by including an expansion of the rich contextual information about each environmental sample.

The question of centralized versus decentralized storage is also one of "who pays for the storage," which is a matter with no simple answer. The US National Center for Biotechnology Information (NCBI) is mandated to store all metagenomic data, however, the sheer volume of data being generated means there is an urgent need for appropriate ways of storing vast amounts of sequences. As the cost of sequencing continues to drop while the cost for analysis and storing remains more or less constant, selection of data storage in either biological (i.e. the sample that was sequenced) or digital form in (de-) centralized archives might be required. Ongoing work and successes in compression of (meta-) genomic data [96], however, might mean that digital information can still be stored cost-efficiently in the near future.

## Conclusion

Metagenomics has benefited in the past few years from many visionary investments in both financial and intellectual terms. To ensure that those investments are utilized in the best possible way, the scientific community should aim to share, compare, and critically evaluate the outcomes of metagenomic studies. As datasets become increasingly more complex and comprehensive, novel tools for analysis, storage, and visualization will be required. These will ensure the best use of the metagenomics as a tool to address fundamental question of microbial ecology, evolution and diversity and to derive and test new hypotheses. Metagenomics will be employed as commonly and frequently as any other laboratory method, and "metagenomizing" a sample might become as colloquial as "PCRing." It is therefore also important that metagenomics be taught to students and young scientists in the same way that other techniques and approaches have been in the past.

## Competing interests

The authors declare that they have no competing interests.

## Authors' contributions

All authors contributed to the conception and writing of the review article. All authors have read and approved the final manuscript.
